# Anniversary Meeting of the Salop Infirmary

**Published:** 1893-11-25

**Authors:** 


### ANNIVERSARY MEETING OF THE SALOP INFIRMARY.

A hundred and forty-six years of useful life is no small age among our provincial hospitals, and Shrewsbury may well feel proud of its institution. As far back as 1737 a scheme was drawn up for the founding of a hospital, and had it been satisfactorily accomplished at that time, the Salop Infirmary would have enjoyed the distinction of being the second oldest provincial hospital in England. Little support was, however, given to the proposal at that time, but in 1745 a site had been selected and building was about to commence, when the defeat of the English troops at Prestonpans threw the whole country into a panic, and the idea was abandoned for the time; but in 1747, the obstacles overcome, we find the hospital opened, with accommodation for 54 patients in the building, and four interns and one extern were at once received. After that date the institution grew and flourished and enlargements were added in 1749, '51, '78, and '88, while in 1830 the demand for admittance was so greatly beyond the accommodation that the old building was pulled down and an entirely new one erected on the same site. The generosity displayed at that time by the people of Shropshire cannot be too highly praised, and for the first 100 years, notwithstanding the disturbed state of Europe, the financial position of the infirmary was extremely satisfactory; the receipts exceeded the expenditure in eighty different years, while from 1808 to 1830 there was always a surplus, the smallest being £125, while in one year it went up to £950. The same generous spirit was displayed in 1847, when, to celebrate the centenary of the institution a chaplaincy was endowed.

Turning to the year completed last Midsummer, we regret to find a certain amount of financial difficulty, so that the unfortunate expedient of utilising legacies for current expenses has been necessitated, and a deficit still remains; but this has been caused by the determination of the governors to spare no expense in securing the efficiency of the charity. Over £100 has been expended in repairs, and the cost of the dispensary has been increased by £250, owing to the number of out-patients exceeding the previous year by 1,826.

After the annual meeting in the Infirmary a procession was formed and marched to St. Mary's Church, where the anniversary service was held. Over fifty clergy were seated in the chancel; the sermon was preached by the Archbishop of York and the Bishop of Shrewsbury, and the Ven. Archdeacon Lloyd read the lessons. The organ was supplemented by a small orchestra, who gave their services gratuitously, and materially added to the beautiful rendering of the choral service. So large was the congregation that many persons were obliged to stand throughout the entire service, and it was gratifying to note that a large proportion of the congregation consisted of members of the county families, who came long distances, braving the inclement November weather, to testify to their interest in their well-beloved Infirmary. A significant token of this practical co-operation may be seen in the old custom of having the most recent bride and youngest *débutante* connected with the county stand at the church door holding plates to receive the offerings from the departing congregation; and in the old days, it is said, the ladies stood in their ball dresses! This costume has long been discontinued, and this year the arrangements were more reverent and decorous. The collection was made in the church by the churchwardens and sidesmen, and given by them to the ladies—the Duchess of Sutherland and the Hon. Miss Bridgeman—who carried it to the entry of the choir, where a clergyman received it and handed it to the Archbishop for presentation. The collection, which was one of the largest ever received, amounted to £296.

